# Dissemination of evidence-based cancer control interventions among Catholic faith-based organizations: results from the CRUZA randomized trial

**DOI:** 10.1186/s13012-016-0430-6

**Published:** 2016-05-18

**Authors:** Jennifer D. Allen, Maria Idalí Torres, Laura S. Tom, Bryan Leyva, Ana V. Galeas, Hosffman Ospino

**Affiliations:** 1 Community Health Program and Department of Public Health and Community Medicine, Tufts University, Medford, MA USA; 2 Dana-Farber Cancer Institute, Boston, MA USA; 3 Mauricio Gaston Institute for Latino Community Development and Public Policy, University of Massachusetts, Boston, MA USA; 4 Warren Alpert Medical School of Brown University, Providence, RI USA; 5 Boston College, Chestnut Hill, MA USA

**Keywords:** Latinos, Hispanics, Faith-based organizations, Catholic, Cancer screening, Evidence-based interventions, Implementation science, Organizational capacity, Capacity building, Community-based participatory research

## Abstract

**Background:**

The CRUZA randomized trial tested the efficacy of an organizational-level intervention to increase the capacity of Catholic faith-based organizations (FBOs) serving Latinos to implement evidence-based strategies (EBS) for cancer control.

**Methods:**

Thirty-one Catholic parishes were enrolled. Twenty were randomized to a “capacity enhancement” (CE) intervention and 11 to a “standard dissemination” (SD) condition. Each received a Program Implementation Manual and Toolkit of materials culturally adapted for FBOs with Latino audiences for five types of EBS recommended by the US Preventive Services Community Guide. CE parishes were offered a menu of capacity-building activities over a 3-month period, while SD parishes were provided a one-time consultation by an Intervention Specialist. Baseline and follow-up surveys compared the number and types of EBS offered.

**Results:**

At baseline, only one parish had offered any cancer-related program in the prior year, yet a third (36 %) had offered some other type of health program or service. At post-intervention follow-up, all parishes offered a greater number of EBS. The only statistically significant difference between CE and SD groups was the number of parishes offering small media interventions (90 % in CE, 64 % in SD; *p* < 0.05).

**Conclusions:**

All parishes increased the number of cancer control activities offered to their members. These findings suggest that Catholic parishes may already have capacity to implement EBS if they are appropriately adapted and packaged and may only require low levels of support to carry out programming. Further research is needed to examine the extent to which program offerings continued after the period of grant funding.

**Trial registration:**

Clinicaltrials.gov NCT01740219.

## Background

Community-based organizations’ (CBOs) adoption and implementation of evidence-based strategies (EBS) for community-based cancer control is essential for translation of research into practice. Cancer control programs delivered by established CBOs are generally more responsive to cultural and linguistic characteristics of the targeted populations and well utilized by community members [1]. However, limited studies have sought to understand existing and needed organizational capacities among CBOs to adopt and implement EBS [2–4]. Moreover, few rigorous trials have evaluated the efficacy of organizational-level strategies, such as technical assistance, training, and mini-grants [2–4], for promoting EBS adoption and implementation among CBOs.

Faith-based organizations (FBOs) are among the most trusted CBOs in Latino communities [5–7], long recommended as venues to reach populations marginalized from the mainstream health care system [8, 9]. Several factors make FBOs natural partners for the delivery of EBS to promote health, including their ubiquity, access to large segments of the population (including medically underserved groups), infrastructures (e.g., health ministries, communication channels), support networks, and personnel resources (e.g., volunteers, lay leaders) [8–10]. Moreover, growing evidence suggests that “faith-based” programs (i.e., rooted in faith and religious teachings), or health interventions with cultural and religious adaptations (e.g., integration of prayer, religious traditions, rituals, writings) [11] may be particularly effective for religious audiences [7, 12, 13].

Studies in the USA show that many FBOs view health as integral to their mission [8], yet only 10 % sponsor any health-related programming [14]. While FBOs as a whole have a predilection for health programming, the low actual sponsorship of health programs suggests the need for interventions to enhance capacity of FBOs to adopt, implement, and sustain EBS for cancer control. Notably, Latino churches provide outstanding access to medically underserved Latino populations but are largely understudied as an organizational setting for cancer control [15–21]. The majority of published interventions in FBOs have been in Black churches [9, 13, 22]. Moreover, prior studies in FBOs have mainly considered strategies and approaches to promote individual-level behavior change [9, 13, 22]. Few studies have tested interventions to promote organizational-level change [23, 24], and of these, none have been among Latino churches. In this context, we designed the CRUZA study to evaluate the efficacy of an organizational-level capacity-enhancement intervention in facilitating implementation of EBS to promote cancer control among Latinos in FBOs.

## Methods

### Study design

CRUZA was a randomized trial with Catholic churches (hereafter referred to as “parishes”) as the unit of randomization and intervention. CRUZA focused on parishes because more than half of Latinos in the USA self-identified as Catholic in 2013 [25], and nearly 50 % of Catholic Latinos over age 50 report attending church one or more times a week [25]. Our goal was to test the efficacy of an intervention designed to build parishes' capacity to implement EBS for cancer control. The primary outcome was the difference in the mean number of EBS for cancer control across intervention conditions between baseline and follow-up. This study was approved by the Institutional Review Boards at the Harvard School of Public Health and the University of Massachusetts, Boston. Study procedures and baseline findings have been described in detail elsewhere [26, 27].

### Community engagement

CRUZA employed a community-based approach, in recognition of the importance of community expertise and engagement in all phases of the research process. Central to our cross-collaborative work was our community advisory committee (CAC), composed of key community stakeholders representing Latino community organizations, local government, cancer advocacy, and regional dioceses. The CAC’s roles are described in detail elsewhere [26], but of relevance to this paper was the active role that CAC members played in contributing information regarding the acceptability and feasibility of various capacity-enhancement interventions. For example, given the challenges with trying to bring groups of representatives from participating CE parishes together for group training, they strongly recommended that we provide individual or on-site technical assistance to each of the sites. The CAC also played a critical role in the process of integrating religious principles and practices into our study messaging. The study’s name—CRUZA—highlights this fact. The Spanish verb “CRUZA” means “to cross” and is symbolic of the study’s cross-collaboration among FBOs, academic institutions, and CBOs. As a cognate of the word cross (*cruz*, in Spanish), “CRUZA” also evokes a clear Christian reference to healing and salvation—a key Catholic tenet.

Extensive formative research preceded the intervention trial, including 18 key informant interviews among Latino faith and community leaders, as well as eight focus groups totaling 67 Spanish-speaking parishioners. These formative research activities illuminated contextually appropriate strategies for engaging faith communities, developing capacity-building interventions, and adapting EBS for delivery in parishes, [5, 6, 28, 29]. This work also informed the types and formats of small media and promotional materials that were included in the tool kit.

### Organizational recruitment, consent and randomization

At the time of study initiation, there were 577 Catholic parishes located in Massachusetts [30]. Prior to parish recruitment, we contacted the four dioceses (administrative units of parishes in a geographic region) in the state, namely Boston, Fall River, Springfield, and Worcester to develop networks of collaboration with their heads (diocesan Bishops) and Hispanic Ministry directors. With the support of the diocesan structures, we utilized a two-stage sampling scheme to identify eligible parishes, and within them, eligible representatives to respond to organizational surveys. Individuals eligible to complete organizational surveys occupied formal roles within the FBO (e.g., pastor, business manager, director of Hispanic Ministry) as indicated by the pastor. Potential respondents received study materials and informed consent information by mail. Informed consent information was also reviewed prior to survey administration. This process is described in detail elsewhere [26, 27], but briefly, parishes that completed a baseline organizational survey between July and December 2012 were invited to participate in the CRUZA intervention trial [26]. Eligible parishes (a) were Roman Catholic; (b) located in Massachusetts; and (c) offered at least one Spanish-language mass per week. In addition, we required that eligible parishes not be scheduled for or undergoing closure or merger at the time of enrollment [31–33]. Prior to enrollment, pastors had to agree to meet or speak with CRUZA staff to review details of the intervention and evaluation and, if randomized to CE group, to designate a parish liaison to work with CRUZA’s Intervention Specialists during the study period. Participating parishes were blocked on size of congregation (≥1500 or <1500 parishioners) and randomized within blocks on a two-to-one ratio to the capacity enhancement (CE) or standard dissemination (SD) conditions using a random number generator. Randomization was conducted by the principal investigator.

### Intervention framework

As an organizational-level intervention, CRUZA applied theoretical principles from the Consolidated Framework for Implementation Research (CFIR). Influences to implementation include (1) the characteristics of the intervention, (2) inner and outer organizational contexts, (3) characteristics of the implementer, and (4) clearly established implementation protocols [34]. Much time and energy were invested in adapting and packaging EBS for a Latino FBO setting, as CFIR suggests that interventions most likely to be utilized by organizations are those that are designed and packaged for the “end user” (“design quality and packaging”), are not overly complicated to implement (“complexity”), can be tried on a small scale (“trialability”) at a low cost, and are modified to meet local needs (“adaptability”). This involved extensive formative research focusing on the relationship between Catholic religious traditions and teachings with health and health behaviors [5, 6, 28, 29].

The CFIR also posits that organizations that have leadership support and engagement, sufficient resources for implementation, and access to the knowledge and skills necessary for adoption/implementation of the innovation (i.e., “readiness for implementation”) are more likely to adopt innovations. Moreover, organizations that have capacity for and collective receptivity to change (i.e., positive “implementation climate”); values consistent with the innovation (i.e., conducive “organizational culture”), and inter-organizational relationships that can facilitate innovation implementation are also more likely to adopt and/or implement innovations [34]. Thus, to implement a new program activity, an organization needs both infrastructure (i.e., policies, procedures, and resources) and the implementers (people with the expertise who will “champion” the program).

### Capacity-enhancement intervention

Several published reviews have shown that skills-based training, technical assistance, and coalition building can enhance organizational capacity for specific innovations [35–38]. Various models for capacity building have been documented [39–48], but most include components such as provision of technical assistance; engagement of organizational members and leaders; partnership development; and training or education to provide information and skills for implementation. The CFIR model speaks directly to the “readiness” of an organization to implement an intervention, positing that leadership engagement (i.e., involvement of and sanction for the intervention from pastors), available resources (e.g., personnel, time, services), and access to information (e.g., Program Manual, Intervention Specialists) are key to successful implementation processes. In addition, the framework recognizes that the characteristics of individuals or groups charged with implementation may play a role in the success of such efforts (e.g., skills, self-efficacy).

In light of existing research, the CFIR model, input from our CAC and key Latino FBO leaders and in consideration of study resources (e.g., funding, study duration, number of CRUZA Intervention Specialists), we designed the CRUZA capacity-enhancement intervention to include (a) technical assistance, (b) formation of health committees or ministries, (c) facilitation of inter-institutional partnerships, and (d) skill-building workshops.

#### Technical assistance

CRUZA Intervention Specialists provided individual guidance to parish liaisons to impart knowledge and skills to implement EBS. Tailored to a parish liaison’s skillset, interests, and communication preferences, technical assistance included coaching, information provision, and/or problem-solving offered in-person meetings, telephone, or email upon request.

#### Health committees

Health ministries are bodies within the parish that plan and execute health-related activities as part of the parish’s overall mission [49]. In parishes with existing health ministries, we offered activities to expand their capacity to plan and execute EBS. In parishes without existing health ministries, CRUZA Intervention Specialists worked with the pastors to identify and recruit potential committee members and plan/facilitate meetings.

#### Inter-institutional partnerships

CRUZA Intervention Specialists facilitated inter-institutional partnerships between parishes and existing community resources such as local and state health departments, community health centers, hospitals, and social service agencies. Examples include brokering a parish’s connections to health insurance navigators, mobile screening vans, and guest speakers for health-related workshops.

#### Skill-building workshops

Led by CRUZA Intervention Specialists, regional “faith and health” were half-day workshops for parish leaders and CRUZA parish liaisons that emphasized Catholic teachings on health and social justice, Latino health disparities, spirituality, and methods for planning and implementing EBS.

This menu of capacity-enhancement (CE) activities were delivered by a team of five bilingual (English/Spanish) CRUZA Intervention Specialists with complementary health education, community advocacy, research, and faith-based experience. Two held master’s degrees (social work and public health respectively), four were actively involved in their own Latino faith-based communities, and all five were trained by the investigators on human subjects research and study protocols and procedures.

### CRUZA’s five evidence-based strategies

Based on recommendations from the US Preventive Services Task Force Community Guide [50], five EBS to promote breast, cervical, colorectal cancer screening included (1) small media; (2) group education; (3) client reminders; (4) reduction of structural barriers to screening; and (5) one-to-one education. To identify components for inclusion in the CRUZA Program Manual and Tool Kit, we thoroughly examined the online archives of Research-Tested Intervention Programs (RTIPS) [51] and Cancer Control P.L.A.N.E.T. (Plan, Link, Act, Network with Evidence-based Tools) [52] for available research-tested intervention protocols under the five EBS. Leading intervention researchers were also identified and subsequently consulted [53, 54] (see Table 1).

**Table 1:** CRUZA evidence-based strategies (EBS) and sample materials

EBS	Community guide definitions^a^	Packaged into CRUZA Toolkit
Small media	Videos and printed materials such as letters, brochures, and newsletters	• Bookmarks • Parish bulletin inserts • Brochures • Tip sheets • Posters • Videos • Magnets
Group education	Presentations, lectures and other interactive formats conducted by health professionals or trained laypeople	• Guest speakers • Meet and learn • CRC Bingo
Client reminders	Written or telephone messages advising people that they are due for screening	• Birthday bulletin inserts • Birthday cards
One-to-one education	Delivery of information by health professionals, lay health advisors, or volunteers by telephone or in-person in medical or community settings	• Conversations after mass
Reducing structural barriers	Facilitating access by addressing non-economic burdens that make it difficult for people to access cancer screening (e.g., distance, time, language)	• Establish partnership with community health center • Volunteers for transportation and childcare

^a^Adapted from Guide to Community Preventive Services. Cancer prevention and control: client-oriented interventions to increase breast, cervical, and colorectal cancer screening. www.thecommunityguide.org/cancer/screening/client-oriented/index.html

Through this process, we identified 28 community intervention programs/protocols designed to increase utilization of breast, cervical, and/or colorectal screening. To be considered as candidates to be included in the CRUZA Program Manual and Toolkit however, each program had to meet two inclusion criteria: (1) cultural and linguistic appropriateness for Spanish-speaking Latinos; and (2) appropriateness for FBOs. As none of the 28 programs identified met both criteria, we leveraged our formative research and community advisory committee expertise and proceeded to adapt and consolidate EBSs following NCI’s “Using What Works” guidelines [55] to meet the above criteria while retaining core elements integral to the internal logic of the intervention programs [56].

EBS materials, all written in English and Spanish at the 6th grade level, were packaged into a user-friendly CRUZA Program Manual and Toolkit. The Program Manual offers a step-by-step activity guide for each EBS along with planning tools, sample materials, and resource guides. The toolkit contains EBS materials for easy distribution in parishes: Bible bookmarks, parish bulletin inserts, spiritually themed photo frames with health messages, birthday cards with reminders about age-appropriate screening guidelines, and bi-fold brochures that weaved family, faith, and health messages [26].

### Intervention conditions

Parishes randomized to the capacity-enhancement (CE) condition received the CRUZA Program Manual and Toolkit, as well as support from CRUZA Intervention Specialists based on a standardized menu of CE activities over a 3-month period of time. Parishes randomized to the standard dissemination (SD) condition received a CRUZA Program Manual and Toolkit; the pastor or designated parish representative in the SD condition was provided with an initial consultation with a CRUZA Intervention Specialist. At the initial consultation meeting (in person or by phone), which lasted between 30 and 60 min, the Intervention Specialist provided instructions for use of the CRUZA Program Manual and Toolkit. This meeting also provided an opportunity for parish representatives to ask questions and to discuss potential barriers to program implementation. While intervention staff did not offer direct assistance with overcoming any of the anticipated barriers, they pointed out materials that were provided in the Program Manual and Toolkit that were designed to help overcome those barriers. Subsequent requests for programmatic assistance from SD parishes during the intervention period were referred to local community resources (e.g., American Cancer Society, community health centers).

### Data collection

#### Organizational surveys

There were four sections of the baseline survey, each varied in the content and intended respondent: (1) part A—leadership (pastor); (2) part B—bookkeeping (business manager); (3) part C—Hispanic ministry (director of Hispanic ministry); and (4) part D—health/social services (parish nurse or director of social outreach). Appropriate respondents for each survey component were identified by the pastor. Trained, bilingual survey assistants who were not involved with intervention administration contacted organizational respondents first by phone. When phone attempts were not successful, additional contact strategies included in-person meetings, mail, and email. Baseline organizational surveys took approximately 60 min to complete (20 min per section) and were conducted between July and December 2012. The follow-up survey, conducted between March and August 2013, took considerably less time (20 min) as there was no need to collect data on stable parish characteristics such as congregation size and composition. A detailed description of recruitment methods, sampling procedures, respondent characteristics, and response/completion rates for organizational assessments is available [27].

#### Process tracking system

A process tracking system tracked the number and types of interventions offered by parishes. Data for this system was collected from the CRUZA liaisons at each parish on a weekly basis by Intervention Specialists. Liaisons provided information about the type of EBS implemented, the date when implemented, and the number of parish participants reached by the EBS. This system was also used to track the CE dosage (e.g., duration, frequency, amount), type of support (e.g., technical assistance, building health ministries, facilitating inter-organizational linkages, skill-building workshops), and delivery mode (e.g., in-person, phone, email) offered by the Intervention Specialists to each parish. Information about program offerings was also obtained as part of the follow-up surveys administered to all participating parishes.

### Measures

#### Primary outcome

The primary outcome was the mean change in the number of EBS for cancer control offered by parishes between baseline and follow-up by intervention condition. This information was gathered through two sources. First, the baseline and follow-up organizational surveys, which took place approximately 1 year apart, included two open-ended questions adapted from a national study of FBOs [14]. The first question asked, “*Has your parish participated in or supported health-related projects or programs of any sort to serve the members of your parish within the past twelve months?*” When the response was “Yes”, a second question was asked, “*What health-related projects or programs has your parish sponsored or participated in within the last 12 months?*” This data was verified through the process tracking system. Second, information was collected by Intervention Specialists on a weekly basis and recorded in the process tracking system, as described above.

#### Parish characteristics

We also assessed parish resources (e.g., size, monetary collections, volunteerism), leader characteristics (e.g., educational level, number of pastoral staff); existing health-related ministries or committees, and existing/prior inter-organizational ties and collaborations with hospitals or health centers.

### Analysis

Our primary hypothesis was that parishes receiving the CE intervention would offer a greater number of EBS for cancer control than those in the SD comparison condition. We had originally intended to evaluate this hypothesis with a two-factor mixed ANOVA, using planned contrasts for the interaction of one *between-subjects* factor (CE vs SD) and one *within-subjects* factor (repeated measures at baseline and follow-up). Sample size calculations were based on assumptions from empirical data that 10 % of FBOs would have offered some form of cancer education program in the prior year and that the intervention would have a small to moderate effect size (0.2–0.4). However, given that only one of the parishes had offered a health program that addressed cancer in the prior year, we were unable to conduct this analysis. Instead, we evaluated differences in the number of EBS at the final survey between intervention conditions with *t* tests and Pearson’s chi-square tests.

## Results

As a requirement for study participation, all parishes had to have completed the baseline survey. Of the 39 parishes that did so (80 %), 34 (87 %) agreed to participate in the CRUZA trial. Of the 34 parishes randomized to intervention conditions, 3 in the CE condition did not meet study requirements (i.e., pastor did not meet with CRUZA study staff or did not complete the final survey). This left an analytic sample of *N* = 31 parishes (20 in CE and 11 in SD). Of the 31 parishes, approximately three quarters had a Hispanic Ministry, though only a quarter had an organized *Health* Ministry. Approximately a third (36 %) had offered some form of health program or service in the prior year. Most reported having established relationships with hospitals or health centers, although this mostly reflected sharing communion, prayer and social support by Catholic priests or lay persons in institutional settings, such as hospitals, nursing homes, and prisons, as opposed to health promotion activities. See Table 2.

**Table 2:** Structural Characteristics of CRUZA parishes by intervention condition, baseline (*n* = 31)

	All parishes mean/% (SD)	Capacity enhancement mean/% (SD)	Standard dissemination mean/% (SD)	*p* value*	*N* (CE/SD)
Parish resources					
Size of congregation	854 (802)	1042 (899)	564 (538)	0.09^a^	17/11
Size of Congregation (sm)^b^	85.7 %	82.4 %	90.9 %	0.53^c^	17/11
Percent of congregation that is Latino	43.0 %	35.5 %	58.3 %	0.34	19/10
Percent of congregation that volunteer	7.0 %	8.4 %	4.7 %	0.36	19/11
Years of Spanish mass offered	20.0 (14.6)	21.6 (16.0)	16.7 (11.6)	0.34	20/10
Parish leadership and staff					
Number of full-time paid pastoral staff	6.2 (8.1)	7.5 (9.7)	3.9 (3.6)	0.24	19/11
Number of full-time non-pastoral staff	7.6 (8.3)	8.9 (9.7)	5.4 (4.4)	0.17	20/11
Percent of pastors with a graduate degree	83.3 %	84.2 %	81.8 %	0.87	19/11
Parish programming: health					
Hispanic Ministry (y)	77.4 %	80.0 %	72.7 %	0.64	20/11
Organized Health Ministry (y)	22.6 %	25.0 %	18.2 %	0.66	20/11
Offered health program in past year (y)	35.5 %	40.0 %	27.3 %	0.48	20/11
Existing Collaborations					
Partnership with hospitals or health centers	86.2 %	89.5 %	80.0 %	0.48	19/10

^a^Independent *t* tests; *α* = 0.05

^b^Congregation size is based on a cut-off value that is either smaller or larger than 1500

^c^Chi-square test; *α* = 0.05

*p-value compares Capacity Enhancement to Standard Dissemination conditions

### Adequacy of randomization procedures in achieving balanced treatment arms

Independent sample *t* tests confirmed that there were no significant differences between the intervention groups with respect to financial resources, pastor’s socio-demographic characteristics, health program offerings, or levels of existing collaborations with health or social service institutions (see Table 2).

### Implementation of EBS for cancer control by intervention condition

The percentage of parishes in each intervention condition that offered each of the five types of EBS is presented in Table 3. During the study period, most parishes offered one or more of the CRUZA EBS. The most commonly offered intervention was small media. CE parishes offered significantly more small media interventions compared with SD parishes (90 vs 64 %, *p* = 0.038). With the exception of reduction of structural barriers, a greater proportion of CE parishes offered three of the other EBS types compared with SD parishes (group education 60 vs 36 %; client reminders 65 vs 55 %; one-to-one educational outreach 70 vs 64 %), although these differences were not statistically significant. Among the CE parishes, 20 % implemented all five types of EBS, while this was only true for 9 % of SD parishes (data not shown). One of the CE parishes did not participate in any of the CRUZA activities, including the initial consultation. When that parish was removed from the analysis, there was a marginally significant (*p* > 0.10) difference in the group education offerings in CE parishes as compared with SD churches (63 vs 36 %).

**Table 3:** Percent of CRUZA parishes that implemented EBS for cancer control by intervention condition, final (*n* = 31)

Strategy	Capacity enhancement (%) (*n* = 20)	Standard dissemination (%) (*n* = 11)	*p* value
Small media^a^	90	64	0.038*
Group education^b^	60	36	0.104
Client reminders^c^	65	55	0.284
Reduction of structural barriers^d^	45	55	0.305
One-to-one education^e^	70	64	0.359

^a^The Z-Score is 1.7777. The *p*-value is 0.03754. The proportion of Yes or No responses for Observation 1 is 0.9. The proportion for Observation 2 is 0.636

^b^The Z-Score is 1.26. The *p*-value is 0.10383. The proportion of Yes or No responses for Observation 1 is 0.6. The proportion for Observation 2 is 0.364

^c^The Z-Score is 0.5718. The *p*-value is 0.28434. The proportion of Yes or No responses for Observation 1 is 0.65. The proportion for Observation 2 is 0.545

^d^The Z-Score is -0.5088. The *p*-value is 0.30503. The proportion of Yes or No responses for Observation 1 is 0.45. The proportion for Observation 2 is 0.545

^e^The Z-Score is 0.3627. The *p*-value is 0.35942.The proportion of Yes or No responses for Observation 1 is 0.7. The proportion for Observation 2 is 0.636

*p-value compares Capacity Enhancement to Standard Dissemination conditions

### Capacity-enhancement dose

Among CE parishes, work with Intervention Specialists to build health committees was the most common activity, with a mean of 11.5 (range 1–37) instances of capacity-building activities offered by CRUZA Intervention Specialists. To a slightly lesser extent, CE parishes engaged in an average of 10.7 contacts for technical assistance (range 0–31). Fewer Intervention Specialist activities were directed toward facilitating inter-institutional relationships (mean 3.7; range 0–23) and just over half of the parishes (60 %) took advantage of the skill-building workshops. See Table 4.

**Table 4:** Number and types of support provided to capacity enhancement (CE) parishes by CRUZA Intervention Specialists, *n* = 19

Type of support	Mean (SD) number of CE activities per parish	Range
Technical assistance	10.7 (8.7)	0–31
Health committee or ministry	11.5 (9.0)	1–37
Inter-organizational relationships	3.8 (7.0)	0–23
	Percentage	
Skill-building workshops	60 %	

## Discussion

To our knowledge, this is first randomized trial of an organizational-level intervention aimed at improving capacity among FBOs to implement EBS for cancer control for Latinos [57]. As such, there are several important lessons to be gleaned from this initiative. First, CRUZA attests to the feasibility of conducting a randomized trial to increase uptake of EBS among Catholic parishes serving Latino populations. Second, it suggests that by packaging and appropriately adapting EBS for the intended audience, Catholic parishes are able to offer EBS for their congregations—even without “Specialist” support. Third, while our findings suggest that these parishes have existing capacity to implement EBS for cancer control, even a brief intervention to support their efforts could increase the number and variety of activities that can be implemented.

Few studies have taken rigorous approaches to designing, describing, and evaluating community-based capacity-building intervention strategies, as supported by a recent systematic review that identified and examined 29 empirical studies of capacity-building interventions conducted between 2000 and 2014 [57]. Most of these studies were conducted in school settings or among community coalitions; most targeted individual behaviors such as drinking and substance abuse, sun exposure, and other youth risk behaviors. Only one study that built organizational capacity targeted cancer screening behaviors—a group non-randomized trial evaluating mini-grants and technical assistance on the implementation of Cancer Control Planet EBS in three community-based organizations [58]. And only one study was conducted in church settings – an evaluation of adoption and implementation of the evidence-based Body and Soul program in six churches using mini-grants plus technical assistance [59]. According to the systematic review, only 12 of the 29 empirical capacity-building studies were group randomized trials—the study designs of the remaining 17 included group non-randomized trials, single group pre-post designs, and case studies. Our randomized trial of a capacity-enhancement intervention for EBS in faith-based settings fills a sizeable gap in this literature.

We found that CE parishes in this study offered significantly more small media interventions compared with SD parishes and a greater proportion of CE parishes offered three of the other EBS compared with SD parishes, albeit this latter finding did not reach statistical significance. Our results are somewhat consistent with previous randomized trials on the efficacy of capacity-building interventions designed to promote adoption and implementation of EBS. A handful of prior studies found non-significant group differences in adoption rates [60–63], while two studies found significantly higher adoption rates in intervention groups than in comparison groups [64, 65]. In the first of the two studies that found higher adoption rates following a capacity-building intervention, the evidence-based Communities That Care (CTC) prevention system for youth substance abuse, delinquency, and other behaviors was implemented and the 12 intervention communities that received technical assistance via telephone calls, email, and annual site visits had significantly higher adoption of CTC programs than the 12 control communities [64]. In the second study, AIDS service organizations receiving a capacity-building package of implementation manuals, staff training workshops, and follow-up consultation resulted in more frequent adoption of evidence-based HIV prevention models [65]. It is important to note that in most capacity-building interventions, technical assistance has typically been proactively provided [34].

Although the CRUZA intervention required that the pastor attend one introductory meeting and designate a liaison to the study, parish liaisons could select the types of support they received from CRUZA Intervention Specialists. Despite our lack of proactive technical assistance and financial incentives, we still found significant increases in uptake of EBS. In CRUZA’s case, we offered a menu of capacity-building components to build the skills of members of health ministries and to connect them to other institutions in the local community, but the use of small media was still more widely adopted than any other EBS. These findings suggest the need for further attention to the development of program characteristics of the other EBS—to increase their appeal or enhance their feasibility of use. Indeed, evidence-based programs with more complex components may be more difficult or require more effort to support [35, 38, 66]. However, it is also possible that differences in receipt of capacity-enhancement support among intervention parishes could explain the variations we observed in parish adoption of EBS for cancer control.

We must acknowledge important limitations of this study. With only 31 parishes, we had limited statistical power to detect differences between groups. Our original power calculations were based on two assumptions that did not hold true (i.e., that 10 % of parishes would have offered some form of cancer-related program or activity in the prior year and that few SD parishes would utilize the CRUZA materials). A post hoc power calculation shows that we would have needed a sizeable increase in the number of parishes in each intervention condition to detect a small to moderate effect size or perhaps a non-intervention control group. Our findings are limited in generalizability as we included only one religious denomination. While more than half of Latinos living in the USA self-identify as Catholic, Latinos are increasingly aligning with non-Catholic and non-Christian churches, which typically have different structures and practices [25, 67]. Additional studies may be needed to extend our findings beyond the Catholic parishes enrolled in this trial. Finally, this study would have been strengthened with additional data collection to examine the issue of sustainability. We do not know if CRUZA parishes were able to sustain the level of activity that they demonstrated during the three-month observation period. For instance, the Archdiocese of Boston’s Office of Health Care Ministry committed to continuing their support of CRUZA and anecdotal evidence among the CE parishes suggests that some EBS activities were maintained beyond the 3-month study period. Nevertheless, we cannot say for certain that this was the case without a more rigorous evaluation of sustainability.

Despite these limitations, this study provides important information about the potential impact of a short-term, organizational-level intervention designed to promote implementation of EBS for cancer control. We have demonstrated the ability to achieve high response and participation rates among Catholic parishes [27]. Moreover, because we had enumerated all eligible churches in MA and had high participation, we have support for the external validity of our findings within the Catholic church. In addition, this study has a number of implications for public health practice and future research. Most EBS have been developed under “ideal” research conditions and are not packaged for easy uptake by FBOs. Existing interventions tend to be accompanied by implementation manuals written for research protocols, not for the lay public [68]. Moreover, many interventions have not been developed with diverse cultural and linguistic audiences in mind, posing challenges for dissemination efforts targeting FBOs and Latino communities. Findings from this study suggest that adapting existing interventions for cultural, linguistic, and setting characteristics and equipping FBOs with an easy-to-follow implementation guide can result in impressive uptake of EBS, especially when coupled with CE efforts (e.g., skills training, workshops, etc.) to boost the capacity of FBOs to implement these strategies. In our study, minimal capacity enhancement (3 months) resulted in meaningful increases in the number and types of EBS implemented by parishes and showcase the promise of working with FBOs, particularly Catholic parishes, for implementing EBS for cancer control in Latino communities.

It is important that we do not understate the effort and time that went into identifying, adapting, and translating existing cancer control EBS for delivery in Latino Catholic parishes. We identified few “ready-made,” “user-friendly” interventions that could be integrated into the CRUZA Program Manual and Toolkit. These gaps in translational research reinforce the call for well-designed public health interventions specifically for diverse audiences, applicable and acceptable for ‘”natural” settings, and scalable without major adaptations or need for high levels of training [69–75].

Further research is also needed to understand the science of capacity building, as it relates to promoting uptake of research-tested interventions for cancer control. The CRUZA trial tested a bundled menu of capacity-enhancement strategies; we did not set out to evaluate which of these components or “ingredients” is most important for enhancing organizational capacity, how these components operate independently and/or together, or to assess the effects of intervention activities on individual-level or group-level competencies. Determining the best ways to impart skills to church lay leaders and volunteers necessary for implementation of EBS may improve the efficiency of future organizational-level interventions.

## Conclusions

Our findings offer encouraging evidence that packaging and appropriately adapting EBS for cancer control can increase implementation of EBS in Catholic parishes and that even a brief organizational-level intervention to enhance parish capacity to could increase the number and variety of EBS activities that can be implemented for cancer control. This research may serve as a foundation for future comparative studies on the potential contribution of faith-based settings to address cancer and other health disparities among Latinos and other immigrant groups.

## Abbreviations

CAC: community advisory committee
CBOs: community-based organizations
CE: capacity enhancement
CFIR: consolidated framework for implementation research
CRUZA: Spanish word that literally means “to cross”. Its symbolic meaning is explained in the paper
CTC: Communities That Care
EBS: evidence-based strategies
FBOs: faith-based organizations
SD: standard dissemination
